# Signalling Properties of Inositol Polyphosphates

**DOI:** 10.3390/molecules25225281

**Published:** 2020-11-12

**Authors:** Tania Maffucci, Marco Falasca

**Affiliations:** 1 Centre for Cell Biology and Cutaneous Research, Blizard Institute, Barts and The London School of Medicine and Dentistry, Queen Mary University of London, London E1 2AT, UK; 2 School of Pharmacy and Biomedical Sciences, CHIRI, Curtin University, Perth 6102, Australia

**Keywords:** inositol phosphates, cell signaling, experimental pharmacology, pleckstrin homology domain, inositol 1,3,4,5,6-pentakisphosphate, inositol 1,2,3,4,5,6-hexakisphosphate

## Abstract

Several studies have identified specific signalling functions for inositol polyphosphates (IPs) in different cell types and have led to the accumulation of new information regarding their cellular roles as well as new insights into their cellular production. These studies have revealed that interaction of IPs with several proteins is critical for stabilization of protein complexes and for modulation of enzymatic activity. This has not only revealed their importance in regulation of several cellular processes but it has also highlighted the possibility of new pharmacological interventions in multiple diseases, including cancer. In this review, we describe some of the intracellular roles of IPs and we discuss the pharmacological opportunities that modulation of IPs levels can provide.

## 1. Introduction

**Figure 1:**
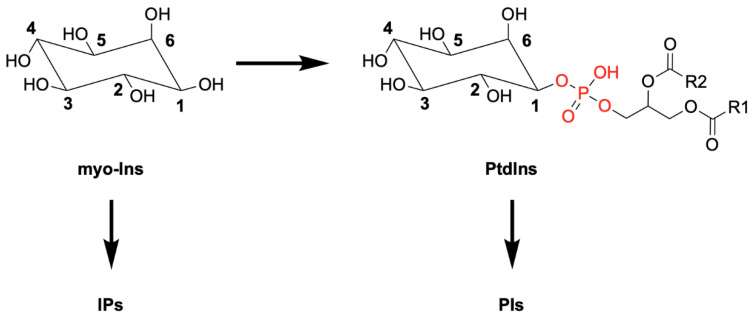
Schematic representation of *myo*-inositol (Ins) and its derivative phosphatidylinositol (PtdIns), showing the attached diacylglycerol linked to the *myo*-Ins head group via a phosphodiester bond (in red). Differential phosphorylation of *myo*-Ins or PtdIns generates the family of inositol polyphosphates (IPs) and phosphoinositides (PIs), respectively.

The water-soluble inositol polyphosphates (IPs) and the inositol lipids phosphoinositides (PIs) are all derivatives of inositol (Ins), especially *myo*-Ins, the most common isomeric form (Figure 1) [1].

*Myo*-Ins can derive from glucose-6-phosphate in a process involving its cyclization to Inositol 3-phosphate (Ins3*P*) or Inositol 1-phosphate (Ins1*P*) (catalyzed by *myo*-inositol-3-phosphate synthase or *myo*-inositol-1-phosphate synthase, respectively), followed by dephosphorylation, catalyzed by inositol monophosphatase. Alternatively, Ins1*P* or Inositol 4-phosphate (Ins4*P*) can derive from dephosphorylation of Inositol 1,4,5-trisphosphate (Ins(1,4,5)*P*_3_) [1]. *Myo*-Ins possesses one axial and five equatorial hydroxyl groups whose differential phosphorylation leads to distinct inositol species (IPs). In fact, over thirty distinct IPs have been described in mammalian cells so far [2], including inositol pyrophosphates (PP-IPs), pyrophosphorylated IPs that derive from further phosphorylation of the “fully phosphorylated” Inositol 1,2,3,4,5,6-hexakisphosphate (Ins*P*_6_) through formation of highly energetic phosphoanhydride bonds (pyrophosphates) at specific positions. Several well-known kinases and phosphatases catalyze the interconversion of IPs, with some showing specificity for selective species, such as Inositol pentakisphosphate 2-kinase (IPPK) which converts Inositol 1,3,4,5,6-pentakisphosphate (Ins*P*_5_) into Ins*P*_6_, and others showing a broader specificity towards different species, such as Inositol polyphosphate multikinase (IPMK). IPMK is essential for the synthesis of both Inositol 1,4,5,6-tetrakisphosphate (Ins(1,4,5,6)*P*_4_) and Inositol 1,3,4,5-tetrakisphosphate (Ins(1,3,4,5)*P*_4_) as well as Ins*P*_5_ [3,4,5,6]. IPMK can also phosphorylate Inositol 1,3,4,6-tetrakisphosphate (Ins(1,3,4,6)*P*_4_) [6]. IPMK is a key enzyme for the synthesis of the higher phosphorylated IPs, as demonstrated by the impaired synthesis of Ins*P*_5_ and Ins*P*_6_ detected in mouse embryonic fibroblasts (MEFs) deficient for the enzyme [7]. Furthermore, MEFs lacking all the three Inositol 1,4,5-trisphosphate 3-kinases (ITPKA, ITPKB and ITPKC) that specifically phosphorylate the 3-phosphate position of the inositol ring still produce Ins*P*_5_ and Ins*P*_6_, confirming that the main synthetic route of these IPs is through IPMK [8]. However, recent studies suggest that ITPKs regulate Ins*P*_6_ synthesis through an alternative route in mammalian cells [9] and that ITPKA plays a critical role in maintaining Ins*P*_6_ cellular levels in mammalian colorectal cancer cell lines [10]. Many inositol products play a crucial role in eukaryotic cells and are involved in a wide number of biochemical functions. This review covers some examples of the processes regulated by IPs and mentions some of the potential pharmacological opportunities arising from modulation of IPs levels. The many roles of PP-IPs have been covered elsewhere in this special issue [11].

## 2. Ins(1,4,5)*P*_3_, a Bona Fide Second Messenger

**Figure 2:**
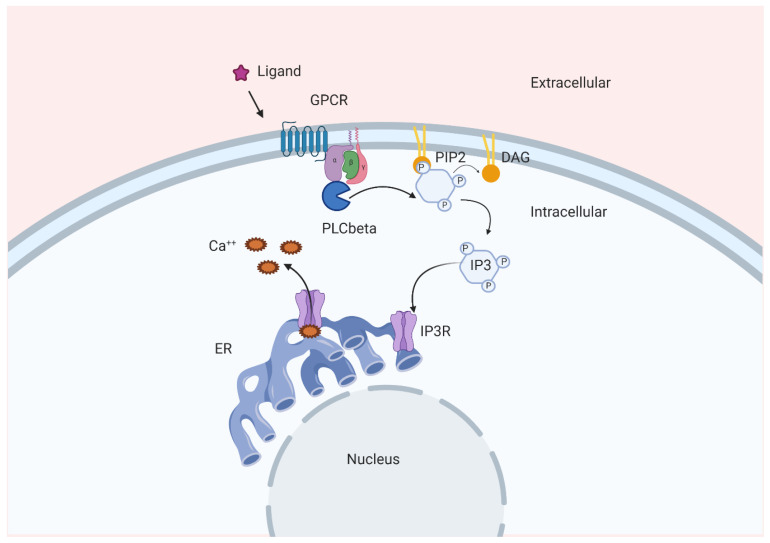
Schematic representation of one of the pathways involved in Ins(1,4,5)P_3_-dependent Ca^2+^ release. GPCR, G protein-coupled receptor; IP3, Ins(1,4,5)*P*_3_; Ca^++^, calcium; ER, endoplasmic reticulum; IP3R, Ins(1,4,5)*P*_3_ receptor; DAG, diacylglycerol; PIP2, PtdIns(4,5)*P*_2_.

Ins(1,4,5)*P*_3_ was the first IP to be identified as a “second messenger” more than 35 years ago [12,13] and its identification as a key regulator of Ca^2+^ release from intracellular stores sparked much interest on the role of inositol-derived molecules in signal transduction. Specifically, Ins(1,4,5)*P*_3_ derives from the hydrolysis of phosphatidylinositol 4,5-bisphosphate (PtdIns(4,5)*P*_2_), which is catalyzed by members of the family of phospholipases C (PLC). Upon cellular stimulation and PLCs activation, the accumulated Ins(1,4,5)*P*_3_ can bind to Ins(1,4,5)*P*_3_ receptors which are expressed in most animal cells and are Ca^2+^ channels located on intracellular organelles such as the endoplasmic reticulum (ER) and Golgi apparatus. Such binding mediates Ca^2+^ release from intracellular stores, resulting in Ca^2+^ accumulation in the cytosol and in organelles such as mitochondria and lysosomes. A schematic representation of the Ins(1,4,5)*P*_3_-mediated Ca^2+^ release following activation of G protein-coupled receptors and of some members of the PLC family is depicted in Figure 2. The resulting decrease in ER luminal Ca^2+^ concentration also triggers stromal interaction molecule 1, which then amasses at ER–plasma membrane (PM) junctions and opens Orai1, a hexameric Ca^2+^ channel in the PM [14,15], causing store-operated Ca^2+^ entry. Through these Ins(1,4,5)*P*_3_-mediated mechanisms, many extracellular stimuli induce redistribution of Ca^2+^ from the ER to the cytosol or other organelles as well as extracellular Ca^2+^ entry [16]. Ca^2+^ regulates a multitude of intracellular processes including transcription, apoptosis, motility, excitability and exocytosis, mainly through modulation of protein conformations and charge [17]. Together with its direct role in signalling, Ins(1,4,5)*P*_3_ serves as a precursor for the synthesis of several highly phosphorylated IPs, including Ins(1,3,4,5)*P*_4_, Ins*P*_5_ and Ins*P*_6_. Importantly, a recent study has identified additional mechanisms that can lead to synthesis of higher phosphorylated IPs, independently from the Ins(1,4,5)*P*_3_ pool derived from the lipid PtdIns(4,5)*P*_2_. Such an alternative route involves phosphorylation of inositol monophosphates (such as the previously mentioned Ins3*P* and Ins1*P*) by the kinase inositol tetrakisphosphate 1-kinase 1 and it depends on the metabolic status of the cells [9]. Whether the higher phosphorylated IPs can all be strictly considered “second messengers” has somehow been a matter of debate [9]. Nonetheless, their importance in regulation of a multitude of cellular processes is unquestionable.

## 3. Intracellular Processes Regulated by IPs Binding to Proteins

A key feature of IPs is their ability to bind to proteins and either contribute to maintain their structural fold or promote/stabilize their assembly into complexes or contribute to their activation, in the case of enzymes. Such binding properties are responsible for most of the intracellular roles that have been attributed to IPs. Indeed, IPs have been found to be involved in regulation of a vast array of very different intracellular processes. Just to mention an example of the diversity of intracellular roles that IPs can have, Ins*P*_6_ has been implicated, amongst many other processes that we will mention later, in: (i) regulation of the activity of the RNA editing enzyme adenosine deaminase that act on RNA (ADAR)2 as well as adenosine deaminases that act on transfer RNA (ADAT)1 [18]; (ii) activation of Bruton’s tyrosine kinase, a Tec-family tyrosine kinase that is essential for B-cell function [19]; (iii) regulation of casein kinase 2 [20], a ubiquitous protein kinase that can phosphorylate over 300 proteins involved in cell growth, development and several other cellular functions; (iv) allosteric regulation of Yersinia outer-protein J effectors, important to control their acetyltransferase activity [21]; (v) ubiquitylation, through regulation of the activity state of cullin-RING E3 ubiquitin ligases [22]; (vi) crotonylation, as indicated by data demonstrating that addition of Ins*P*_6_ to the complex histone deacetylase (HDAC)1/CoREST/Lysine specific demethylase 1 increased its initial rate of decrotonylation by 1.8-fold [23].

In fact, IPs have been detected in several X-ray crystal structures and this suggests their involvement in many cellular processes [24]. As additional evidence of these interactions accumulates, the variety of cellular processes that relies on the correct balance between different IPs species is becoming more evident. Few examples of some of the first cellular functions that were attributed to IPs together with some of the most recently identified will be mentioned here. Attention is focused on processes that can provide examples of the rationale behind the design of potential therapeutic strategies based on exploitation of the role of IPs. 

### 3.1. Endo and Exocytosis

The identification of proteins able to bind to Ins*P*_6_, such as clathrin assembly proteins (AP)-2 [25] and AP-3 [26] and arrestin [27] suggested very early a role for this IP in endocytosis and exocytosis. Indeed, Ins*P*_6_ was proposed to be involved in the regulation of both processes in pancreatic β cells, where it appeared to be the most abundant IP [28]. Specifically, Ins*P*_6_ was shown to regulate insulin exocytosis by enhancing the activity of the voltage-dependent Ca^2+^ channels through deactivation of Serine/Threonine protein phosphatases and activation of Serine/Threonine kinases [28,29]. In addition, Ins*P*_6_ was also reported to be involved in the regulation of calcineurin-dependent endocytosis in β cells [30]. Over the years, roles for different IPs, in particular PP-IPs, in the complex process of insulin secretion have been described more precisely [31,32]. A wider role for Ins*P*_6_ in regulation of endocytosis was further indicated by data supporting its involvement in regulation of this process in synaptic vesicle. This was suggested by the identification of its role in phosphorylation of a protein kinase responsible for regulation of the synaptic vesicle-associated protein pacsin/syndapin I and the demonstration that such Ins*P*_6_-regulated phosphorylation increased the interaction between dynamin and pacsin/syndapin I [33]. Importantly, a specific role for Ins*P*_6_ (and isomers of the PP-IP Ins*P*_7_) was reported in this study, with no phosphorylation detected using lower IPs, in particular Ins*P*_3_ or Ins*P*_4_ [33].

### 3.2. Nuclear Functions

Ins(1,4,5,6)*P*_4_ has emerged as a key regulator of some members of the family of class I HDACs, enzymes that regulate lysine acetylation in histone tails and are involved in epigenetic regulation of genes [34]. The role of Ins(1,4,5,6)*P*_4_ was first suggested by its presence in the crystal structure of HDAC3 and its co-repressor silencing mediator of retinoic acid and thyroid hormone receptor (SMRT), specifically in a binding pocket at the interface between the enzyme and the co-repressor [35]. Later studies corroborated that the inositol phosphate-binding pocket was present in other class I HDAC co-repressor complexes, and, indeed, it was reported that Ins(1,4,5,6)*P*_4_ enhances the deacetylase activity of both the HDAC3:SMRT and HDAC1:SMRT complexes [36]. The stereochemical requirement for binding and activation by IPs has also been described [37]. Furthermore, mutations that abolish Ins*P*_4_ binding decrease the activity of HDAC1/2 in vivo [38]. These data indicate a role for Ins(1,4,5,6)*P*_4_ in histone acetylation and chromatin condensation and contribute to the growing interest around the role of IPs and PIs in epigenetic regulation.

A role for Ins*P*_6_ in the nucleus has been suggested by many lines of evidence, including the identification of a well-ordered Ins*P*_6_ molecule in the crystal structure of sister chromatid cohesion protein Pds5 homolog B (Pds5B), a subunit of the cohesin complex, which controls transcription, chromosome segregation and DNA repair [39]. Additional studies have indicated that Ins*P*_6_ has a role in DNA repair, in particular non-homologous end-joining mediated by DNA-dependent protein kinase [40] and nuclear mRNA export [41], acting together with nucleoporin Gle1 [42,43]. A role for Ins*P*_5_ and Ins*P*_6_ in chromatin remodelling was also reported [44,45].

### 3.3. Platelet Aggregation

A recent study has reported the increase of Ins*P*_6_ in platelets upon stimulation with thrombin, collagen I and ADP [46]. Importantly, this study has revealed a role for Ins*P*_6_ in regulation of platelet aggregate size, in a mechanism involving its interaction with fibrinogen. Although previous evidence identified both Ins*P*_5_ and Ins*P*_6_ as binding partners of fibrinogen [47], InsP_5_ did not have a role in regulation of aggregation, which appeared specific for InsP_6_ [46]. The authors suggested a role for Ins*P*_6_ in supporting and stabilizing the crosslinking between fibrinogen and platelets, identifying this IP as a novel potential player in regulation of platelet functions [46]. 

### 3.4. Reactive Oxygen Species (ROS) Formation and Drug Sensitivity

A very recent study reported that Ins*P*_4_ selectively binds to the enzyme NADPH oxidase 4 (NOX4) which is important for ROS generation [48]. Such a direct interaction competes with NADPH binding to NOX4 and inhibits the enzyme, resulting in reduced ROS production. Importantly, the authors showed that downregulation of ITPKB, sensitized cisplatin-resistant cancer cells to cisplatin treatment both in vitro and in vivo. This was likely due to the reduced levels of Ins*P*_4_, which would result in increased ROS production and therefore increased cisplatin-induced ROS production [48]. Consistent with this, the authors reported that expression of ITPKB and cisplatin resistance positively correlated in 22 human cancer cell lines and 13 patient-derived xenograft tumours of head and neck squamous cell carcinoma (HNSCC), lung cancer and ovarian cancer. In addition, it was shown that expression of the enzyme was higher in primary tumours from HNSCC patients who had recurrent disease compared with tumours from responsive patients [48]. The observation that chemical inhibition of ITPKB also sensitized cells to cisplatin treatment both in vitro and in vivo confirms how manipulation of IPs levels can have an important therapeutic value [49].

### 3.5. Viral Replication

**Figure 3:**
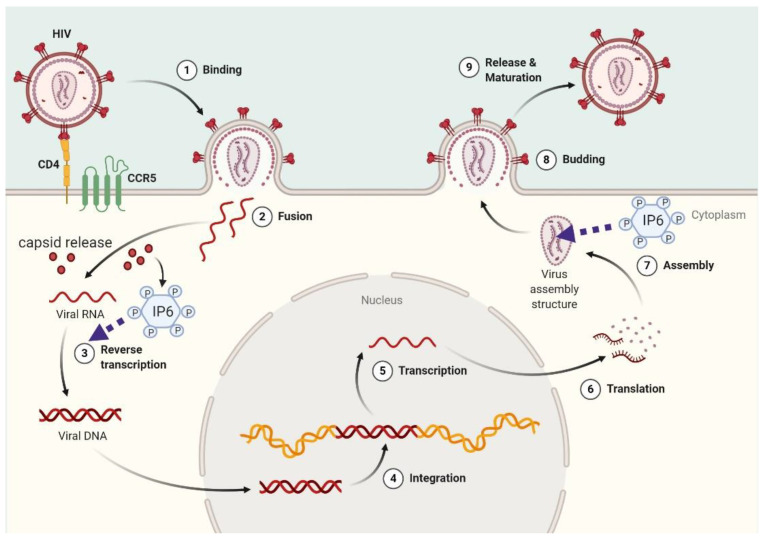
Mechanistic model for Ins*P*_6_ functions in HIV-1 infection. After virus infection, capsids are released into the cytoplasm and Ins*P*_6_ maintains capsid integrity. Before the budding phase, Ins*P*_6_ is recruited in assembling virions.

Several lines of evidence indicate that Ins*P*_6_ plays a crucial role during HIV-1 infection, being involved in some of the several capsid transformations that occur during viral replication. Specifically, it has been reported that HIV-1 recruits Ins*P*_6_ into virions using two Lysine rings in its immature hexamers [50] and that Ins*P*_6_ in turn promotes the assembly and maturation of the mature capsid lattice [51,52,53]. Furthermore, binding of Ins*P*_6_ increases HIV-1 capsid stability from minutes to hours, enabling freshly synthesized DNA to gather inside the capsid during reverse transcription [54]. These data indicate that Ins*P*_6_ is important in HIV-1 assembly and during viral entry by stabilizing the capsid while moving towards the nucleus, making it a critical cofactor for HIV replication (Figure 3).

Indeed, cells lacking IPPK, the enzyme that catalyzes the addition of the final phosphate on the position 2 of Ins*P*_5_, produced much fewer infectious HIV-1 particles [51]. Importantly, however, infectious particles were still being produced in these cells albeit at a much-reduced rate [51]. In line with this, a recent study revealed that, in the absence of Ins*P*_6_, as achieved upon genetic knockout of IPMK and IPPK, HIV-1 packages Ins*P*_5_ and it can substitute Ins*P*_5_ for Ins*P*_6_ during viral production without affecting its infectivity [50]. Indeed, Ins*P*_5_ was found to be able to stimulate immature HIV-1 assembly in vitro, although less efficiently that Ins*P*_6_ [50,51,54]. A very recent study has demonstrated that the almost complete ablation of both Ins*P*_6_ and Ins*P*_5_ induced a 1000-fold reduction (i.e., an almost abrogation) in the production of HIV-1 infectious particle and virus release, establishing an absolute requirement for these IPs in HIV-1 viral production [55]. Furthermore, the authors demonstrated that ablation of Ins*P*_6_ and Ins*P*_5_ in viral target cells did not affect permissivity to HIV-1 infection [55]. Importantly, recent evidence suggests a conserved role for Ins*P*_6_ in lentiviral assembly, as indicated by its ability to stimulate the in vitro assembly of immature particles of many other retroviruses in the lentivirus genus [56]. On the other hand, ablation of IPPK only modestly reduced the production of infectious particles by other retroviruses, such as a gammaretrovirus, a betaretrovirus and two non-primate lentiviruses. This study in particular demonstrated that only the primate (macaque) lentivirus simian immunodeficiency virus displayed a similar dependence on Ins*P*_6_/Ins*P*_5_ as HIV-1 [55].

## 4. Exploiting the Binding Properties of IPs for Therapeutic Purposes

### 4.1. Exogenous IPs 

**Figure 4:**
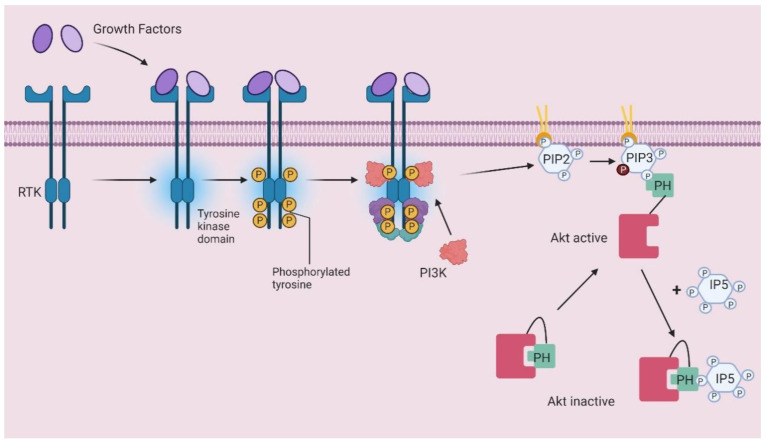
Schematic representation of PIP3-dependent Akt translocation and Ins*P*_5_ competition mechanism. RTK, receptor tyrosine kinase; PIP2, PtdIns(4,5)*P*_2_; PIP3, PtdIns(3,4,5)*P*_3_; PI3K, Phosphoinositide 3-kinase; IP5, inositol pentakisphosphate; PH pleckstrin homology domain.

Until now, the therapeutic potential of IPs has been mainly tested through administration of exogenous IPs. In some cases, this strategy aims to inhibit PIs-dependent signaling pathways through competition between exogenous IPs and endogenous PIs towards same protein domains. The best example of this is provided by proteins containing pleckstrin homology (PH) domains, protein modules that can bind to IPs and PIs [57,58,59]. Twenty years ago, we hypothesized that water soluble IPs could be delivered intracellularly to compete with PIs for PH domain binding and therefore they could inhibit activation of proteins that rely on PIs/PH domain interaction [2]. Over the following years, we and others demonstrated that this strategy was correct and it held potential therapeutic value. Work in our lab was mostly focused on Akt PH domain, whose interaction with phosphatidylinositol 3,4,5-trisphosphate (PtdIns(3,4,5)*P*_3_) is critical for its translocation to the plasma membrane and enzymatic activation. (Figure 4).

As it was known that Ins*P*_5_ and, to a slightly lesser extent, Ins(1,4,5,6)*P*_4_, were also able to bind Akt PH domain [57], we hypothesized that these IPs could compete with PtdIns(3,4,5)*P*_3_ for Akt PH domain binding, resulting in inhibition of Akt translocation to the plasma membrane. A schematic representation of the mechanism of Akt activation upon growth factors-mediated activation of receptor Tyrosine kinases as well as the inhibitory role of exogenous IPs (in particular of Ins*P*_5_) are shown in Figure 4. Indeed, supporting our hypothesis of a potential competition between the two groups of compounds, we observed that plasma membrane translocation of a Green fluorescent protein-tagged Akt PH domain was inhibited by exogenous Ins(1,4,5,6)*P*_4_ [60]. Later additional evidence of such an inhibition appeared, including data showing that a cell permeant Ins(1,3,4,5)*P*_4_ was able to inhibit a receptor-dependent plasma membrane translocation of AKT PH domain in neutrophils [61]. More important, impairment of Akt plasma membrane translocation resulted in inhibition of its activation, as indicated by our subsequent studies showing that exogenously added Ins*P*_5_ inhibited phosphorylation of Akt in ovarian cancer line SKOV-3 cells [62], in PTEN^-/-^ embryonic stem cells [62], in human umbilical vein endothelial cells (HUVEC) activated with basic fibroblast growth factor (FGF-2) [63] and in dissected tumours from a xenograft model of SKOV-3 cells [63]. Notably, our studies confirmed that the IPs-mediated inhibition of Akt phosphorylation was able to affect Akt-dependent cellular processes. Specifically, the addition of exogenous Ins(1,4,5,6)*P*_4_ and Ins*P*_5_ resulted in inhibition of proliferation/growth of breast cancer cell line MCF7, small cell lung cancer cell line H69 and SKOV-3 cells [60]. Furthermore, Ins*P*_5_ induced apoptosis in H69 cells and SKOV-3 cells [62] and inhibited FGF-2-induced cell survival, cell migration and capillary tube formation in HUVECs as well as inhibiting angiogenic response in vivo [63]. Additional evidence of the anti-angiogenic properties of Ins*P*_5_ has been provided by a recent study reporting that Ins*P*_5_ can also inhibit vascular endothelial growth factor production by fibroblasts and neural cells in a mechanism involving degradation of hypoxia inducible factor 1α [64]. The definite demonstration that such a strategy could represent an important potential therapeutic strategy came from the observation that Ins*P*_5_ reduced tumour growth in a xenograft model of SKOV-3 cells [63]. Interestingly, in this experimental setting, treatment with Ins*P*_6_ did not reduce tumour growth or Akt activation in dissected tumours [63].

The potential beneficial effects of exogenous IPs in cancer might go beyond their inhibition of PIs-mediated processes. For instance, Ins*P*_6_ has been reported to have anti-cancer activity in several in vitro and in vivo models [65], including prostate [66,67,68], breast [69,70], bladder [71] and colon [72,73] cancer. Some very preliminary clinical results are also appearing [65]. Whether Ins*P*_6_ exerts its anti-cancer activity by interfering with PIs-mediated activation of proteins is not established, as it has been reported that its inhibition of cancer cell proliferation, cancer cell survival and metastasis [64,74,75] is a result of inhibition of several pathways, including the phosphoinositide 3-kinase [PI3K)/Akt pathway [67,73,76]. The impact of Ins*P*_6_ can be very diverse: recently, for instance, its involvement in regulation of microRNA levels, in particular miR-155, in human colon cancer cells has also been reported [77], suggesting additional mechanisms by which this IP might exert its anti-cancer activity. Indeed, Ins*P*_6_ therapeutic potential has been mainly related to its anti-oxidant properties and its ability to oppose many carcinogens [65]. It must be noted that concentrations of exogenously added Ins*P*_6_ reported to have anti-cancer activity in vitro were usually in the millimolar range (1–5 mM), i.e., concentrations that can induce metals chelation and changes in the pH in cellular medium. To avoid this problem, a recent study has reported the synthesis of a prodrug of Ins*P*_6_ which retains the pro-apoptotic activity in vitro as well as anti-cancer activity in vivo in a mouse model of adult T-cell leukemia [78]. Whether the anti-cancer activity is due to Ins*P*_6_ itself or to its rapid conversion to other IPs, namely Ins*P*_5_ and Ins*P*_4_, remains a matter of debate. Analysis of the IPs in extracts of intact HeLa cells incubated with ^3^H-Ins*P*_6_ revealed that ^3^H-Ins*P*_3_, ^3^H-Ins*P*_4_ and ^3^H-Ins*P*_5_ accumulated inside the cells, confirming that internalized Ins*P*_6_ was dephosphorylated into lower forms [79]. Moreover, these authors reported that both Ins(1,4,5,6)*P*_4_ and Ins*P*_5_ were more active than Ins*P*_6_ in inducing apoptosis [79]. Consistent with this, recent evidence indicated that Ins*P*_6_ derivatives, resulting from partial degradation of ingested Ins*P*_6_ by phytase into hydrolysates, also possess anti-cancer activity. For instance, it has been reported that partially degraded Ins*P*_6_ inhibited proliferation of HCT116 colon carcinoma cells [80]. Similarly, a study demonstrated that Ins*P*_6_ hydrolysates inhibited proliferation of the colorectal cancer cell line SW620, with evidence suggesting that Ins*P*_4_ and Ins*P*_5_ were the likely primary constituents of the hydrolysates responsible for the anti-proliferative activity via Akt inhibition [72]. In contrast to Ins*P*_6_, we found that exogenous Ins*P*_5_ not only was quickly and systematically internalized by cells but it was also converted slowly into different metabolites [63], supporting the conclusion that its anti-tumour effects were due to its activity and were not mediated by conversion to different phosphorylated forms.

Potential beneficial effects of exogenous IPs have also been explored in other diseases including diabetes [81], which, possibly, is not surprising considering that IPs and PP-IPs have been involved in insulin secretion, as previously mentioned.

### 4.2. Interfering with IPs/Proteins Binding

As we discussed above, many of the cellular roles of IPs depend on their ability to bind proteins and modulate their assembly into complexes or their activation. This raises the interesting possibility of inhibiting selective cellular processes by modulating intracellular levels of IPs through blockade of one of the enzymes responsible for their interchange or by developing compounds that might interfere with the selective IPs/proteins binding. For instance, small molecules might be designed to interfere with the recruitment of Ins*P*_6_ in the immature hexamers or possibly compete with Ins*P*_6_ and therefore they might block HIV viral particles production. Similar compounds might interfere with Ins*P*_6_ to fibrinogen and therefore possibly be beneficial to destabilize thrombi, as hypothesized recently [46].

However, even though this strategy is potentially interesting, possible limitations need to be taken into accountthe use of IPs as novel therapeutics should consider the variety of roles that they have in cell homeostasis, such as insulin signaling and nuclear processes [82], that could potentially generate off-target effects and hence side effects.

## 5. Conclusions

The family of IPs has been implicated in a multitude of intracellular functions and new evidence constantly appears identifying their contribution to additional processes. Such a variety and versatility of roles mean that several opportunities exist to exploit these molecules from a therapeutic point of view, either by delivering IPs and/or their analogues intracellularly or by manipulating the enzymes that control their interchanges or by interfering with their interaction with their effector proteins. In this respect, additional studies to shed light into the specific mechanisms of interaction between IPs and their effector proteins (such as X-ray crystal structure and modelling) might provide important information to design novel strategies to interfere with their cellular processes.

## References

[1] Michell R.H. (2008). Inositol derivatives: Evolution and functions. Nat. Rev. Mol. Cell Biol..

[2] Berrie C.P., Falasca M. (2000). Patterns within protein/polyphosphoinositide interactions provide specific targets for therapeutic intervention. FASEB J..

[3] Resnick A.C. (2008). Inositol polyphosphate multikinase: Metabolic architect of nuclear inositides. Front. Biosci..

[4] Odom A.R., Stahlberg A., Wente S.R., York J.D. (2000). A Role for Nuclear Inositol 1,4,5-Trisphosphate Kinase in Transcriptional Control. Science.

[5] Nalaskowski M.M., Deschermeier C., Fanick W., Mayr G.W. (2002). The human homologue of yeast ArgRIII protein is an inositol phosphate multikinase with predominantly nuclear localization. Biochem. J..

[6] Chang S.-C., Miller A.L., Feng Y., Wente S.R., Majerus P.W. (2002). The Human Homolog of the Rat Inositol Phosphate Multikinase Is an Inositol 1,3,4,6-Tetrakisphosphate 5-Kinase. J. Biol. Chem..

[7] Frederick J.P., Mattiske D., Wofford J.A., Megosh L.C., Drake L.Y., Chiou S.-T., Hogan B.L.M., York J.D. (2005). An essential role for an inositol polyphosphate multikinase, Ipk2, in mouse embryogenesis and second messenger production. Proc. Natl. Acad. Sci. USA.

[8] Leyman A., Pouillon V., Bostan A., Schurmans S., Erneux C., Pesesse X. (2007). The absence of expression of the three isoenzymes of the inositol 1,4,5-trisphosphate 3-kinase does not prevent the formation of inositol pentakisphosphate and hexakisphosphate in mouse embryonic fibroblasts. Cell. Signal..

[9] Desfougères Y., Wilson M.S.C., Laha D., Miller G.J., Saiardi A. (2019). ITPK1 mediates the lipid-independent synthesis of inositol phosphates controlled by metabolism. Proc. Natl. Acad. Sci. USA.

[10] Dovey C.M., Diep J., Clarke B.P., Hale A.T., McNamara D.E., Guo H., Brown N.W., Cao J.Y., Grace C.R., Gough P.J. (2018). MLKL Requires the Inositol Phosphate Code to Execute Necroptosis. Mol. Cell.

[11] Lee S., Kim M.G., Ahn H., Kim S. (2020). Inositol Pyrophosphates: Signaling Molecules with Pleiotropic Actions in Mammals. Molecules.

[12] Berridge M.J. (1983). Rapid accumulation of inositol trisphosphate reveals that agonists hydrolyse polyphosphoinositides instead of phosphatidylinositol. Biochem. J..

[13] Berridge M.J. (1984). Inositol trisphosphate and diacylglycerol as second messengers. Biochem. J..

[14] Hou X., Pedi L., Diver M.M., Long S.B. (2012). Crystal structure of the calcium release-activated calcium channel Orai. Science.

[15] Yen M., Lewis R.S. (2018). Physiological CRAC channel activation and pore properties require STIM1 binding to all six Orai1 subunits. J. Gen. Physiol..

[16] Prole D.L., Taylor C.W. (2019). Structure and Function of IP3 Receptors. Cold Spring Harb. Perspect. Biol..

[17] Clapham D.E. (2009). A STIMulus Package puts orai calcium channels to work. Cell.

[18] Macbeth M.R., Schubert H.L., VanDeMark A.P., Lingam A.T., Hill C.P., Bass B.L. (2005). Inositol Hexakisphosphate Is Bound in the ADAR2 Core and Required for RNA Editing. Science.

[19] Wang Q., Vogan E.M., Nocka L.M., Rosen C.E., Zorn J.A., Harrison S.C., Kuriyan J. (2015). Autoinhibition of Bruton’s tyrosine kinase (Btk) and activation by soluble inositol hexakisphosphate. eLife.

[20] Lee W.-K., Son S.H., Jin B.-S., Na J.-H., Kim S.-Y., Kim K.-H., Kim E.E., Yu Y.G., Lee H.H. (2013). Structural and functional insights into the regulation mechanism of CK2 by IP6 and the intrinsically disordered protein Nopp140. Proc. Natl. Acad. Sci. USA.

[21] Zhang Z.-M., Ma K.-W., Yuan S., Luo Y., Jiang S., Hawara E., Pan S., Ma W., Song J. (2016). Structure of a pathogen effector reveals the enzymatic mechanism of a novel acetyltransferase family. Nat. Struct. Mol. Biol..

[22] Scherer P.C., Ding Y., Liu Z., Xu J., Mao H., Barrow J.C., Wei N., Zheng N., Snyder S.H., Rao F. (2016). Inositol hexakisphosphate (IP6) generated by IP5K mediates cullin-COP9 signalosome interactions and CRL function. Proc. Natl. Acad. Sci. USA.

[23] Kelly R.D.W., Chandru A., Watson P.J., Song Y., Blades M., Robertson N.S., Jamieson A.G., Schwabe J.W.R., Cowley S.M. (2018). Histone deacetylase (HDAC) 1 and 2 complexes regulate both histone acetylation and crotonylation in vivo. Sci. Rep..

[24] Blind R.D. (2020). Structural analyses of inositol phosphate second messengers bound to signaling effector proteins. Adv. Biol. Regul..

[25] Voglmaier S.M., Keen J.H., Murphy J.-E., Ferris C.D., Prestwich G.D., Snyder S.H., Theibert A.B. (1992). Inositol hexakisphosphate receptor identified as the clathrin assembly protein AP-2. Biochem. Biophys. Res. Commun..

[26] Norris F.A., Ungewickell E., Majerus P.W. (1995). Inositol hexakisphosphate binds to clathrin assembly protein 3 (AP-3/AP180) and inhibits clathrin cage assembly in vitro. J Biol Chem..

[27] Palczewski K., Pulvermüller A., Buczylko J., Gutmann C., Hofmann K.P. (1991). Binding of inositol phosphates to arrestin. FEBS Lett..

[28] Larsson O. (1997). Inhibition of Phosphatases and Increased Ca^2+^ Channel Activity by Inositol Hexakisphosphate. Science.

[29] Efanov A.M., Zaitsev S.V., Berggren P.-O. (1997). Inositol hexakisphosphate stimulates non-Ca^2+^-mediated and primes Ca^2+^-mediated exocytosis of insulin by activation of protein kinase C. Proc. Natl. Acad. Sci. USA.

[30] Høy M., Efanov A.M., Bertorello A.M., Zaitsev S.V., Olsen H.L., Bokvist K., Leibiger B., Leibiger I.B., Zwiller J., Berggren P.-O. (2002). Inositol hexakisphosphate promotes dynamin I- mediated endocytosis. Proc. Natl. Acad. Sci. USA.

[31] Barker C.J., Berggren P.-O. (2013). New Horizons in Cellular Regulation by Inositol Polyphosphates: Insights from the Pancreaticβ-Cell. Pharmacol. Rev..

[32] Rajasekaran S.S., Kim J., Gaboardi G.-C., Gromada J., Shears S.B., Dos Santos K.T., Nolasco E.L., Ferreira S.D.S., Illies C., Köhler M. (2018). Inositol hexakisphosphate kinase 1 is a metabolic sensor in pancreatic β-cells. Cell. Signal..

[33] Hilton J.M., Plomann M., Ritter B., Modregger J., Freeman H.N., Falck J.R., Krishna U.M., Tobin A.B. (2001). Phosphorylation of a Synaptic Vesicle-associated Protein by an Inositol Hexakisphosphate-regulated Protein Kinase. J. Biol. Chem..

[34] De Ruijter A.J., Van Gennip A.H., Caron H.N., Kemp S., Van Kuilenburg A.B. (2003). Histone deacetylases (HDACs): Characterization of the classical HDAC family. Biochem. J..

[35] Watson P.J., Fairall L., Santos G.M., Schwabe J.W.R. (2012). Structure of HDAC3 bound to co-repressor and inositol tetraphosphate. Nat. Cell Biol..

[36] Millard C.J., Watson P.J., Celardo I., Gordiyenko Y., Cowley S.M., Robinson C.V., Fairall L., Schwabe J.W.R. (2013). Class I HDACs Share a Common Mechanism of Regulation by Inositol Phosphates. Mol. Cell.

[37] Watson P.J., Millard C.J., Riley A.M., Robertson N.S., Wright L.C., Godage H.Y., Cowley S.M., Jamieson A.G., Potter B.V.L., Schwabe J.W.R. (2016). Insights into the activation mechanism of class I HDAC complexes by inositol phosphates. Nat. Commun..

[38] Jamaladdin S., Kelly R.D.W., O’Regan L., Dovey O.M., Hodson G.E., Millard C.J., Portolano N., Fry A.M., Schwabe J.W.R., Cowley S.M. (2014). Histone deacetylase (HDAC) 1 and 2 are essential for accurate cell division and the pluripotency of embryonic stem cells. Proc. Natl. Acad. Sci. USA.

[39] Ouyang Z., Zheng G., Tomchick D.R., Luo X., Yu H. (2016). Structural Basis and IP6 Requirement for Pds5-Dependent Cohesin Dynamics. Mol. Cell.

[40] Hanakahi L.A., Bartlet-Jones M., Chappell C., Pappin D., West S.C. (2000). Binding of Inositol Phosphate to DNA-PK and Stimulation of Double-Strand Break Repair. Cell.

[41] York J.D. (1999). A Phospholipase C-Dependent Inositol Polyphosphate Kinase Pathway Required for Efficient Messenger RNA Export. Science.

[42] Miller A.L., Suntharalingam M., Johnson S.L., Audhya A., Emr S.D., Wente S.R. (2004). Cytoplasmic Inositol Hexakisphosphate Production Is Sufficient for Mediating the Gle1-mRNA Export Pathway. J. Biol. Chem..

[43] Alcázar-Román A.R., Tran E.J., Guo S., Wente S.R. (2006). Inositol hexakisphosphate and Gle1 activate the DEAD-box protein Dbp5 for nuclear mRNA export. Nat. Cell Biol..

[44] Shen X., Xiao H., Ranallo R., Wu W.-H. (2002). Modulation of ATP-Dependent Chromatin-Remodeling Complexes by Inositol Polyphosphates. Science.

[45] Steger D.J., Haswell E.S., Miller A.L., Wente S.R., O’Shea E.K., Oòshea E.K. (2002). Regulation of Chromatin Remodeling by Inositol Polyphosphates. Science.

[46] Brehm M.A., Klemm U., Rehbach C., Erdmann N., Kolšek K., Lin H., Aponte-Santamaría C., Gräter F., Rauch B.H., Riley A.M. (2019). Inositol hexakisphosphate increases the size of platelet aggregates. Biochem. Pharmacol..

[47] Grint T., Riley A.M., Mills S.J., Potter B.V.L., Safrany S.T. (2012). Fibrinogen—A Possible Extracellular Target for Inositol Phosphates. Messenger.

[48] Pan C., Jin L., Wang X., Li Y., Chun J., Boese A.C., Li D., Kang H., Zhang G., Zhou L. (2019). Inositol-triphosphate 3-kinase B confers cisplatin resistance by regulating NOX4-dependent redox balance. J. Clin. Investig..

[49] Erneux C., Ghosh S., Koenig S. (2016). Inositol(1,4,5)P 3 3-kinase isoenzymes: Catalytic properties and importance of targeting to F-actin to understand function. Adv. Biol. Regul..

[50] Mallery D.L., Faysal K.R., Kleinpeter A., Wilson M.S., Vaysburd M., Fletcher A.J., Novikova M., Böcking T., Freed E.O., Saiardi A. (2019). Cellular IP6 Levels Limit HIV Production while Viruses that Cannot Efficiently Package IP6 Are Attenuated for Infection and Replication. Cell Rep..

[51] Dick R.A., Zadrozny K.K., Xu C., Schur F.K.M., Lyddon T.D., Ricana C.L., Wagner J.M., Perilla J.R., Ganser-Pornillos B.K., Johnson M.C. (2018). Inositol phosphates are assembly co-factors for HIV-1. Nat. Cell Biol..

[52] Dick R.A., Mallery D.L., Vogt V.M., James L.C. (2018). IP6 Regulation of HIV Capsid Assembly, Stability, and Uncoating. Viruses.

[53] Yu A., Lee E.M.Y., Jin J., Voth G.A. (2020). Atomic-scale characterization of mature HIV-1 capsid stabilization by inositol hexakisphosphate (IP6). Sci. Adv..

[54] Mallery D.L., Márquez C.L., McEwan W.A., Dickson C.F., Jacques D.A., Anandapadamanaban M., Bichel K., Towers G.J., Saiardi A., Böcking T. (2018). IP6 is an HIV pocket factor that prevents capsid collapse and promotes DNA synthesis. eLife.

[55] Ricana C.L., Lyddon T.D., Dick R.A., Johnson M.C. (2020). Primate lentiviruses require Inositol hexakisphosphate (IP6) or inositol pentakisphosphate (IP5) for the production of viral particles. PLoS Pathog..

[56] Dick R.A., Xu C., Morado D.R., Kravchuk V.O., Ricana C.L., Lyddon T.D., Broad A.M., Feathers J.R., Johnson M.C., Vogt V.M. (2020). Structures of immature EIAV Gag lattices reveal a conserved role for IP6 in lentivirus assembly. PLoS Pathog..

[57] Takeuchi H., Kanematsu T., Misumi Y., Sakane F., Konishi H., Kikkawa U., Watanabe Y., Katan M., Hirata M. (1997). Distinct specificity in the binding of inositol phosphates by pleckstrin homology domains of pleckstrin, RAC-protein kinase, diacylglycerol kinase and a new 130 kDa protein. Biochim. Biophys. Acta (BBA) Mol. Cell Res..

[58] Razzini G., Ingrosso A., Brancaccio A., Sciacchitano S., Esposito D.L., Falasca M. (2000). Different subcellular localization and phosphoinositides binding of insulin receptor substrate protein pleckstrin homology domains. Mol. Endocrinol..

[59] Lemmon M.A., Ferguson K.M., O’Brien R., Sigler P.B., Schlessinger J. (1995). Specific and high-affinity binding of inositol phosphates to an isolated pleckstrin homology domain. Proc. Natl. Acad. Sci. USA.

[60] Razzini G., Berrie C.P., Vignati S., Broggini M., Mascetta G., Brancaccio A., Falasca M. (2000). Novel functional PI 3-kinase antagonists inhibit cell growth and tumorigenicity in human cancer cell lines. FASEB J..

[61] Jia Y., Subramanian K.K., Erneux C., Pouillon V., Hattori H., Jo H., You J., Zhu D., Schurmans S., Luo H.R. (2007). Inositol 1,3,4,5-Tetrakisphosphate Negatively Regulates Phosphatidylinositol-3,4,5- Trisphosphate Signaling in Neutrophils. Immunity.

[62] Piccolo E., Vignati S., Maffucci T., Innominato P.F., Riley A.M., Potter B.V.L., Pandolfi P.P., Broggini M., Iacobelli S., Innocenti P. (2004). Inositol pentakisphosphate promotes apoptosis through the PI 3-K/Akt pathway. Oncogene.

[63] Maffucci T. (2005). Inhibition of the Phosphatidylinositol 3-Kinase/Akt Pathway by Inositol Pentakisphosphate Results in Antiangiogenic and Antitumor Effects. Cancer Res..

[64] Fu M., Song Y., Wen Z., Lu X., Cui L. (2016). Inositol Hexaphosphate and Inositol Inhibit Colorectal Cancer Metastasis to the Liver in BALB/c Mice. Nutrients.

[65] Vucenik I. (2019). Anticancer Properties of Inositol Hexaphosphate and Inositol: An Overview. J. Nutr. Sci. Vitaminol..

[66] Agarwal C., Dhanalakshmi S., Singh R.P., Agarwal R. (2004). Inositol Hexaphosphate Inhibits Growth and Induces G1 Arrest and Apoptotic Death of Androgen-Dependent Human Prostate Carcinoma LNCaP Cells1. Neoplasia.

[67] Gu M., Roy S., Raina K., Agarwal C., Agarwal R. (2009). Inositol hexaphosphate suppresses growth and induces apoptosis in prostate carcinoma cells in culture and nude mouse xenograft: PI3K-Akt pathway as potential target. Cancer Res..

[68] Raina K., Ravichandran K., Rajamanickam S., Huber K.M., Serkova N.J., Agarwal R. (2012). Inositol hexaphosphate inhibits tumor growth, vascularity, and metabolism in TRAMP mice: A multiparametric magnetic resonance study. Cancer Prev. Res..

[69] Tantivejkul K., Vucenik I., Eiseman J., Shamsuddin A.M. (2003). Inositol hexaphosphate (IP6) enhances the anti-proliferative effects of adriamycin and tamoxifen in breast cancer. Breast Cancer Res. Treat..

[70] Vucenik I., Ramakrishna G., Tantivejkul K., Anderson L.M., Ramljak D. (2005). Inositol hexaphosphate (IP6) blocks proliferation of human breast cancer cells through a PKCdelta-dependent increase in p27Kip1 and decrease in retinoblastoma protein (pRb) phosphorylation. Breast Cancer Res Treat..

[71] Kandzari S.J., Riggs D., Jackson B., Luchey A., Oliver C., Zaslau S. (2013). In vitro regulation of cell growth and angiogenesis by inositol hexaphosphate in bladder cancer. Curr. Urol..

[72] Chen C., Yang F., Liu C., Cui L., Fu M., Song Y. (2017). Inositol hexaphosphate hydrolysate competitively binds to AKT to inhibit the proliferation of colon carcinoma. Oncol. Rep..

[73] Kapral M., Wawszczyk J., Jesse K., Paul-Samojedny M., Kuśmierz D., Węglarz L. (2017). Inositol Hexaphosphate Inhibits Proliferation and Induces Apoptosis of Colon Cancer Cells by Suppressing the AKT/mTOR Signaling Pathway. Molecules.

[74] Vucenik I., Shamsuddin A.M. (2003). Cancer Inhibition by Inositol Hexaphosphate (IP6) and Inositol: From Laboratory to Clinic. J. Nutr..

[75] Singh R.P., Agarwal R. (2003). Inositol hexaphosphate inhibits growth, and induces G1 arrest and apoptotic death of prostate carcinoma DU145 cells: Modulation of CDKI-CDK-cyclin and pRb-related protein-E2F complexes. Carcinogenesis.

[76] Liu G., Song Y., Cui L., Wen Z., Lu X. (2015). Inositol hexaphosphate suppresses growth and induces apoptosis in HT-29 colorectal cancer cells in culture: PI3K/Akt pathway as a potential target. Int. J. Clin. Exp. Pathol..

[77] Kapral M., Wawszczyk J., Węglarz L. (2019). Regulation of MicroRNA-155 and Its Related Genes Expression by Inositol Hexaphosphate in Colon Cancer Cells. Molecules.

[78] Masunaga T., Murao N., Tateishi H., Koga R., Ohsugi T., Otsuka M., Efujita M. (2019). Anti-cancer activity of the cell membrane-permeable phytic acid prodrug. Bioorganic Chem..

[79] Ferry S., Matsuda M., Yoshida H., Hirata M. (2002). Inositol hexakisphosphate blocks tumor cell growth by activating apoptotic machinery as well as by inhibiting the Akt/NFkappaB-mediated cell survival pathway. Carcinogenesis.

[80] Ishizuka S., Saitoh K.-I., Suzuki T., Lee J.-S., Hara H. (2011). A partially degraded product of phytate suppresses the proliferation of HCT116 colorectal cancer cells. Food Chem..

[81] Omoruyi F.O., Stennett D., Foster S., Dilworth L.L. (2020). New Frontiers for the Use of IP6 and Inositol Combination in Treating Diabetes Mellitus: A Review. Molecules.

[82] Tsui M.M., York J.D. (2010). Roles of inositol phosphates and inositol pyrophosphates in development, cell signaling and nuclear processes. Adv. Enzym. Regul..

